# Maculopathy Linked to Light Chain Monoclonal Gammopathy: A Case Report

**DOI:** 10.1155/crop/2299385

**Published:** 2026-08-21

**Authors:** Christina Mitsi, Doukas Dardabounis, Lydia Inglezou, Konstantinos Liapis, Georgios Labiris, Tryfon Rotsos

**Affiliations:** ^1^ Department of Ophthalmology, University Hospital of Alexandroupolis, Alexandroupolis, Greece, pgna.gr; ^2^ Department of Hematology, Democritus University of Thrace Medical School, Alexandroupolis, Greece, duth.gr

## Abstract

Monoclonal gammopathies can produce a spectrum of ocular manifestations, most commonly paraproteinemic keratopathy, but retinal involvement, though rare, may be vision‐threatening. We report a case of paraproteinemic maculopathy in a 49‐year‐old female with a history of hypertension and albuminuria, who presented with blurred vision and renal impairment. Examination revealed subretinal yellow infiltrates in the posterior pole with hyperautofluorescence on fundus autofluorescence and subretinal fluid with hyperreflective deposits above the retinal pigment epithelium on optical coherence tomography. Laboratory workup identified an excess of free kappa light chains in serum. A diagnosis of light chain deposition disease (LCDD) was made on the basis of a renal biopsy, and systemic therapy was initiated. At 5 months posttreatment, best‐corrected visual acuity improved to 20/20 OU, with resolution of subretinal fluid, although sub‐RPE deposits persisted. This case illustrates that monoclonal free light chains can induce maculopathy through RPE and choroidal depositions, mimicking other macular diseases. Early recognition and systemic treatment targeting the underlying plasma cell disorder are essential for visual recovery. Ophthalmologists should maintain a high index of suspicion for paraproteinemic maculopathy in patients with atypical serous macular detachments, particularly when the findings are bilateral or associated with subtle systemic signs, emphasizing the need for a multidisciplinary approach.

## 1. Introduction

Although rare, monoclonal gammopathies can produce a spectrum of ocular findings through deposition of immunoglobulins in ocular tissues or hyperviscosity‐related vascular effects. The most well recognized ocular involvement in plasma cell dyscrasias is paraproteinemic keratopathy, a corneal deposition of monoclonal immunoglobulin, often seen as crystalline or patchy stromal opacities that is typically associated with monoclonal gammopathy of undetermined significance (MGUS) or myeloma [1]. However, involvement of the retina and choroid is also documented and can be vision‐threatening. In particular, the term “paraproteinemic maculopathy” refers to an exudative macular detachment associated with systemic monoclonal gammopathy. This entity is characterized by serous (exudative) retinal detachments or subretinal fluid (SRF) accumulation in the macula, sometimes with yellowish subretinal deposits, occurring in the setting of an M‐protein in the circulation. This has been observed most often in Waldenström′s macroglobulinemia but also in multiple myeloma and MGUS [2, 3]. In this case report, we present an unusual case of maculopathy linked to light chain deposition disease (LCDD).

## 2. Case Presentation

**Figure 1:**
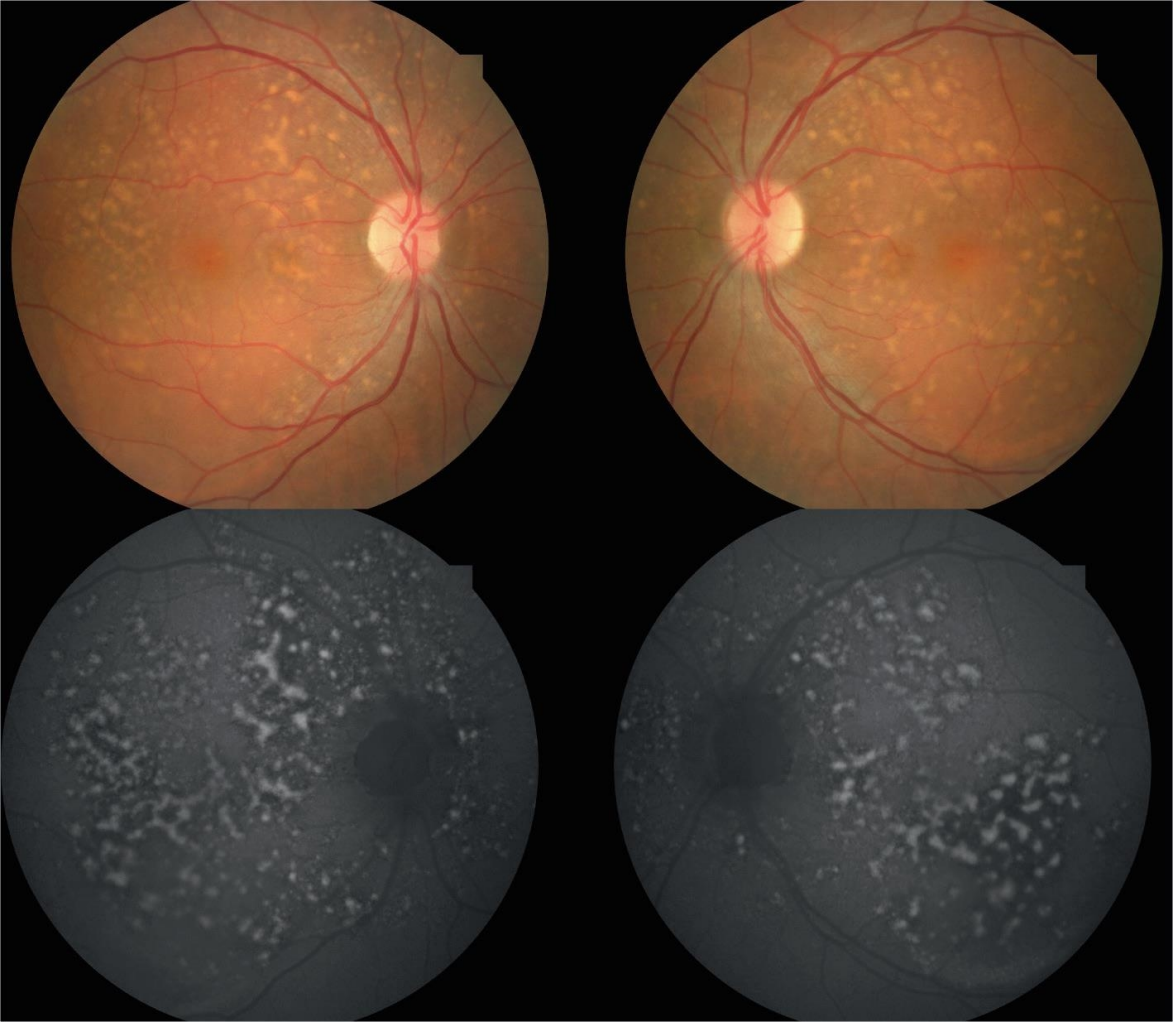
Color and FAF pictures.

**Figure 2:**
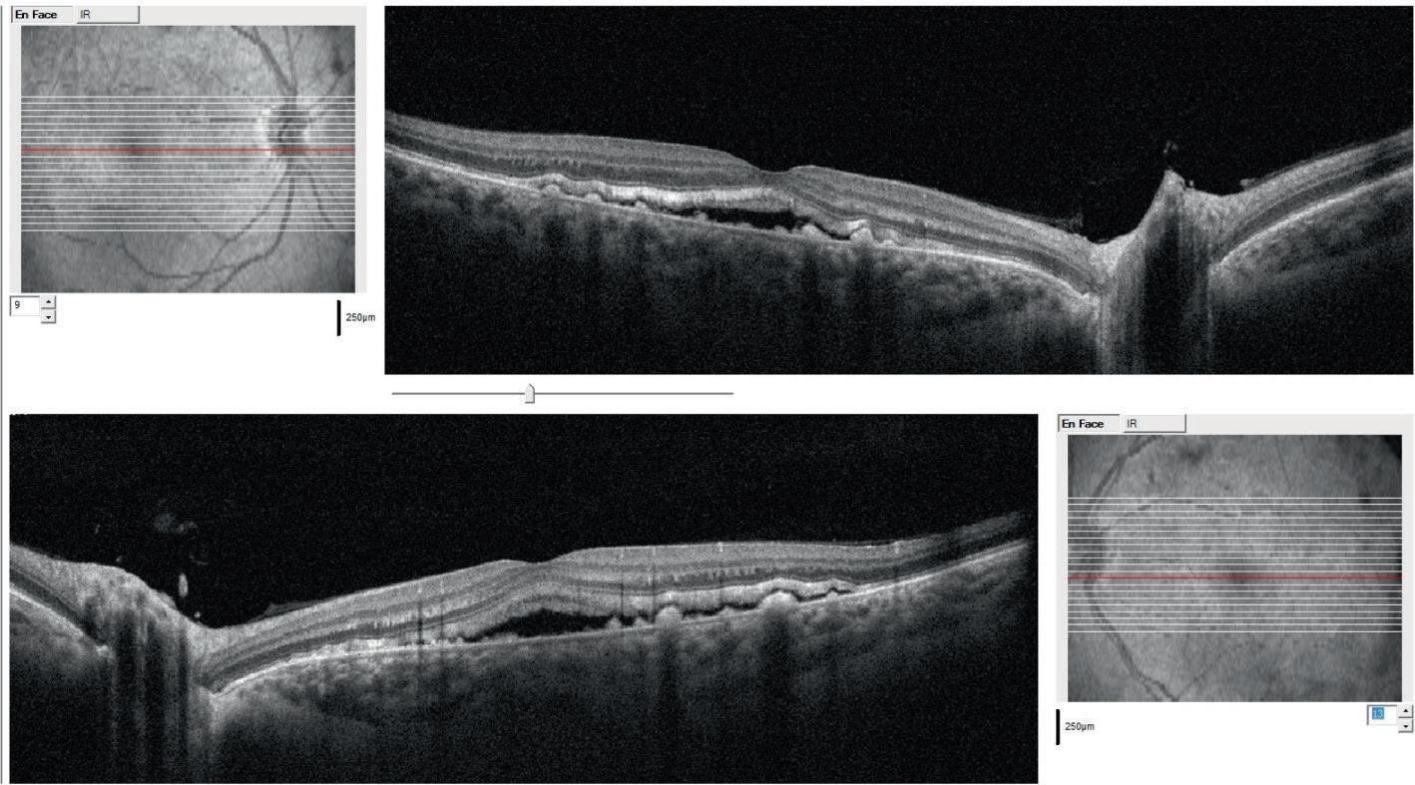
OCT with subretinal fluid and hyperreflective deposits above the RPE.

A 49‐year‐old woman was referred by her nephrologist to our ophthalmology clinic, because of an 1‐week history of blurred vision. She had a history of hypertension and glomerulonephritis, diagnosed 5 years earlier, for which she had received treatment with corticosteroids; her baseline serum creatinine level was 3.95 mg/dL and estimated glomerular filration rate (eGFR) 13 mL/min/1.73 m^2^. Her medical history also consisted of Hashimoto′s thyroid disease. Upon initial examination, best corrected visual acuity (BCVA) was 20/32 OU and there were no significant findings in the anterior segments. On dilated fundus examination, subretinal yellow infiltrates were observed in the posterior pole that were hyperfluorescent in fundus autofluorescence (FAF) (Figures 1 and 2). The differential diagnosis proved challenging; however, due to the age of the patient, the symptom onset, and the morphology of the lesions, primary retinal disorders such as age‐related macular degeneration or autosomal recessive bestrophinopathy were ruled out. Although topical treatment with nepafenac and dorzolamide hydrochloride was initially considered, the distinctive appearance of the lesions led to the suspicion for a systemic disease and the clinicians chose to observe the patient until further investigations were made.

**Figure 3:**
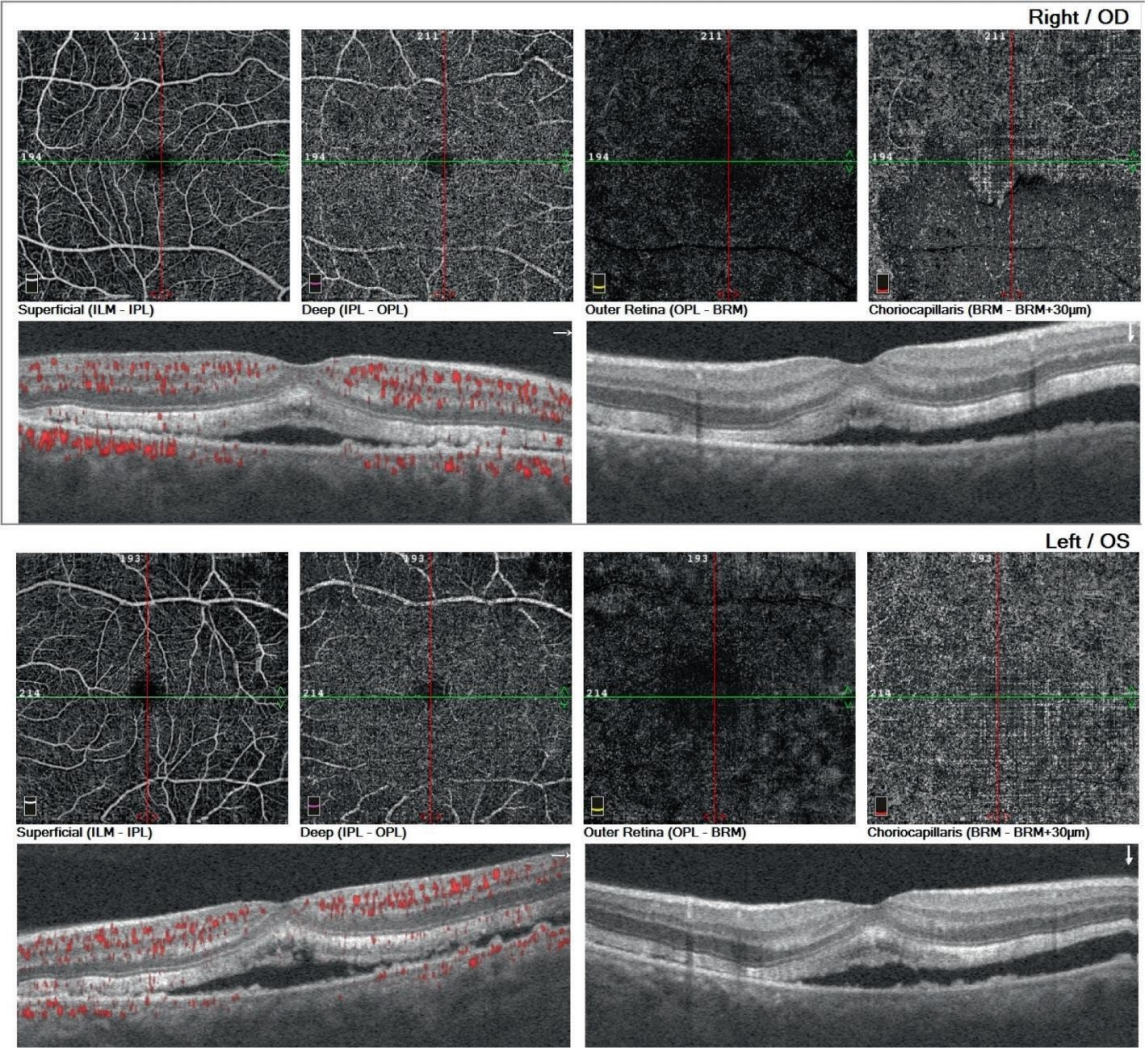
OCT‐A without neovascularization.

Optical coherence tomography (OCT) revealed SRF and hyperreflective deposits above the RPE. However, there were no signs of neovascularization or other pathology in the choroid on the OCT‐A (Figure 3).

After the ophthalmology consult, the patient was referred for extensive laboratory and pathology examinations. After 3 weeks, specialized hematological examinations, bone marrow, and renal biopsies established the diagnosis of LCDD. The patient was found to have nephrotic‐range proteinuria (4.2 g per 24 h), and no monoclonal immunoglobulin was detected in serum or urine. However, free kappa light chains were 522 mg/L in serum (reference range, 8.3–27.0), with lambda light chains 23.94 mg/L (8.3–27.0), and free kappa:lambda ratio 21.83 (0.26–1.65). Bone marrow biopsy showed 8%–10% kappa‐restricted plasma cells, and fluorescent in situ hybridization (FISH) was positive for t(11;14) chromosomal translocation. Treatment with the combination of daratumumab with VCd (bortezomib, cyclophosphamide, and dexamethasone) was initiated.

**Figure 4:**
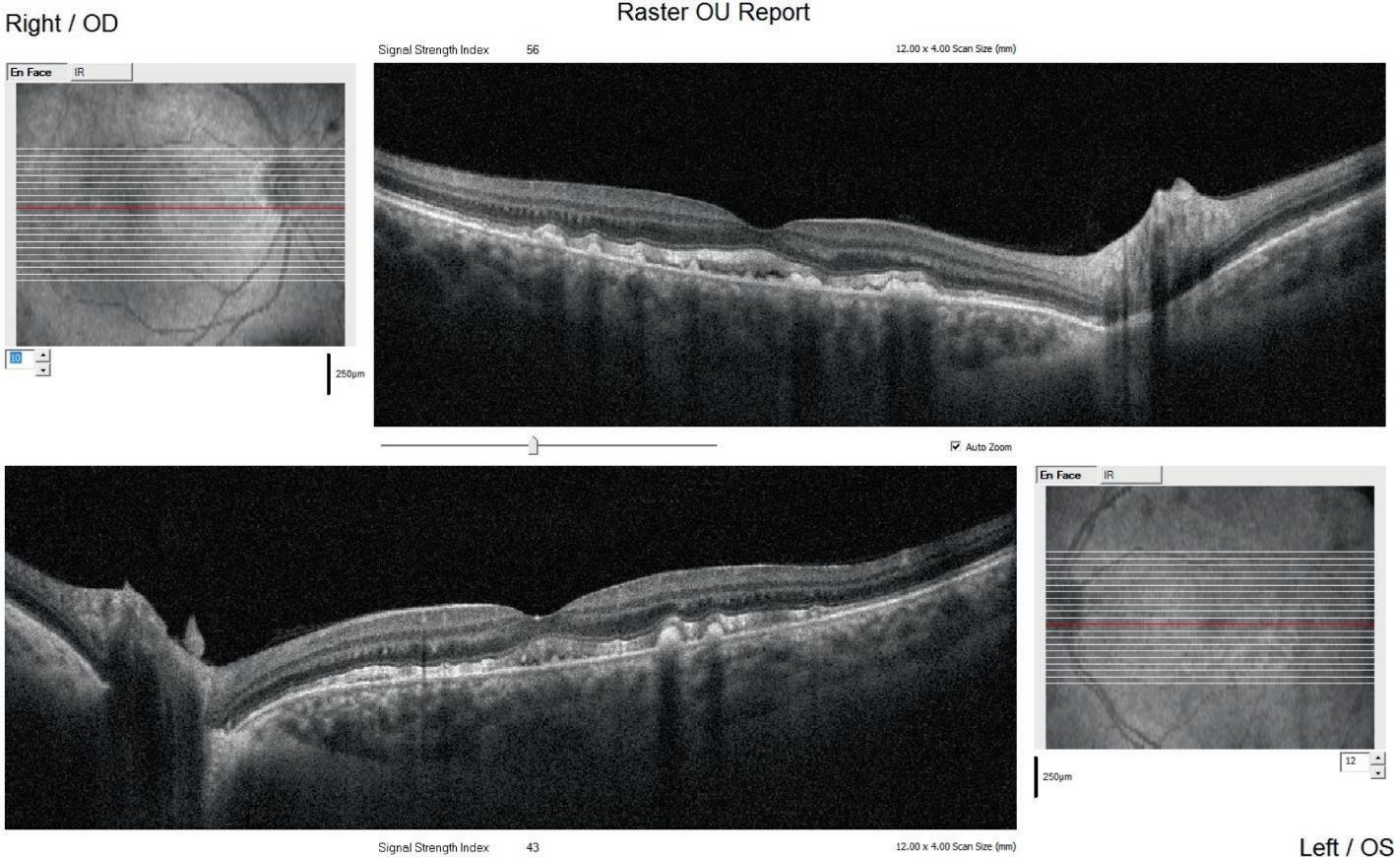
OCT showing significant SRF reduction.

At 5 months after the initiation of systemic treatment, BCVA was 20/20 OU with significant SRF reduction (Figure 4), although the sub‐RPE deposits remained in fundus examination.

## 3. Discussion

MGUS is a premalignant plasma cell disorder characterized by a monoclonal protein (“M‐protein”) in the blood without signs of end‐organ damage. [4]. The risk of progression of MGUS to a malignant plasma cell dyscrasia is well‐recognized. Notably, light‐chain monoclonal gammopathy of undetermined significance (LC‐MGUS) tends to have a higher rate of progression compared to conventional MGUS. When kidney injury, attributable to the monoclonal protein, is present despite a low tumor burden, the term monoclonal gammopathy of renal significance (MGRS) is used. Recently, when ocular tissues are the primary site of injury, authors have proposed the term monoclonal gammopathy of ocular significance (MGOS) for patients with MGUS who have vision‐threatening ocular findings in the absence of other end‐organ damage [5]. LCDD is caused by a plasma cell proliferative disorder in the bone marrow (MGUS or multiple myeloma) that secretes an abnormal kappa light chain, which deposits in tissues, causing organ dysfunction. As many as 96% of patients with LCDD present with renal manifestations (nephrotic syndrome, hypertension, and renal failure). Symptomatic extrarenal deposition, which is uncommon in LCDD, may involve the heart (in 21% of cases), liver, lung, skin, peripheral nervous system, and rarely the ocular fundus, as in our patient. Sub‐RPE deposits in monoclonal gammopathy are believed to result from circulating monoclonal immunoglobulins—most often light chains—escaping from the choroidal circulation, accumulating within the RPE–Bruch′s membrane complex because of abnormal biochemical properties and impaired clearance, and subsequently causing RPE dysfunction and fluid accumulation. An increasing number of reports have described patients with monoclonal gammopathies presenting with serous macular detachments or vitelliform‐like subretinal deposits. Mansour et al. [2] reported a multicenter case series of 33 eyes with paraproteinemic maculopathy (10 new cases and 23 from literature). These patients had underlying diagnoses including Waldenström macroglobulinemia, multiple myeloma, and MGUS. All cases showed neurosensory retinal detachments in the macula with SRF on OCT, often accompanied by subretinal yellowish deposits. The maculopathy was sometimes bilateral but could also be unilateral. Similarly, an earlier series by Rusu et al. [6] described four patients who developed acquired vitelliform macular detachments in the setting of immunoglobulin disorders. In all four, fundus exam revealed an accumulation of yellow submacular material with serous elevation of the retina, and multimodal imaging confirmed subretinal hyperreflective deposits with fluid on OCT and hyperautofluorescent spots on autofluorescence. More recent evidence comes from Zhu et al. [7] who documented bilateral retinal pigment epithelial (RPE) light chain deposition in patients with systemic LCDD. They reported three patients, all of whom showed globular deposits along the RPE–Bruch′s membrane on OCT with associated serous macular detachments. FAF in these cases revealed multifocal hyperautofluorescent spots corresponding to the sub‐RPE deposits, and ICGA/OCT angiography confirmed that the detachments were “angiographically silent” (i.e., not due to classic choroidal neovascular leakage). These findings strongly implicate direct deposition of monoclonal free light chains in the submacular region as a cause of exudative maculopathy. A similar case where multimodal imaging showed progressing pigmented and hyperautofluorescent drusenoid deposits lead to systemic evaluation revealed nephrotic‐syndrome–related renal failure, and the diagnosis of AL amyloidosis was set [8].

## 4. Conclusion

In summary, although uncommon, monoclonal free light chains can cause a paraproteinemic maculopathy through RPE and choroidal infiltration or through secondary hemodynamic effects. The result is a collection of SRF and deposits that can closely mimic more common macular diseases. Given that paraproteinemic maculopathy is mainly caused by the circulating monoclonal protein, the primary treatment goal is to reduce the monoclonal immunoglobulin level and treat the underlying plasma cell dyscrasia. Patients should receive chemotherapy directed at their hematologic condition, as lowering the systemic paraprotein load is far more effective in resolving the maculopathy than any focal ocular treatment and significant anatomical and visual recovery is often achievable. Ophthalmologists should maintain a high index of suspicion for paraproteinemic maculopathy in patients with atypical serous detachments, especially if bilateral or associated with subtle signs of plasma cell dyscrasia, and promptly involve the hematology team for a multidisciplinary approach.

## Funding

No funding was received for this manuscript.

## Consent

Written consent was obtained from the patient for publication of this case report.

## Conflicts of Interest

The authors declare no conflicts of interest.

## Data Availability

Research data are not shared.
